# Activation of SsoPK4, an Archaeal eIF2α Kinase Homolog, by Oxidized CoA

**DOI:** 10.3390/proteomes3020089

**Published:** 2015-05-15

**Authors:** William K. Ray, Mark B. Potters, January D. Haile, Peter J. Kennelly

**Affiliations:** 1 Department of Biochemistry, Virginia Polytechnic Institute & State University, Blacksburg, VA 24061, USA; E-Mails: wkray@vt.edu (W.K.R.); mpotters@vt.edu (M.B.P.); 2 Department of Chemistry, Centre College, Danville, KY 40422, USA; E-Mail: january.haile@centre.edu

**Keywords:** protein phosphorylation, stress signaling, crenarchaea

## Abstract

The eukaryotic protein kinase (ePK) paradigm provides integral components for signal transduction cascades throughout nature*.* However, while so-called typical ePKs permeate the *Eucarya* and *Bacteria,* atypical ePKs dominate the kinomes of the *Archaea*. Intriguingly, the catalytic domains of the handful of deduced typical ePKs from the archaeon *Sulfolobus solfataricus* P2 exhibit significant resemblance to the protein kinases that phosphorylate translation initiation factor 2α (eIF2α) in response to cellular stresses. We cloned and expressed one of these archaeal eIF2α protein kinases, SsoPK4. SsoPK4 exhibited protein-serine/threonine kinase activity toward several proteins, including the *S. solfataricus* homolog of eIF2α, aIF2α. The activity of SsoPK4 was inhibited *in vitro* by 3ʹ,5ʹ-cyclic AMP (K_i_ of ~23 µM) and was activated by oxidized Coenzyme A, an indicator of oxidative stress in the *Archaea.* Activation enhanced the apparent affinity for protein substrates, K_m_, but had little effect on V_max_. Autophosphorylation activated SsoPK4 and rendered it insensitive to oxidized Coenzyme A.

## 1. Introduction

Covalent phosphorylation constitutes nature’s most versatile and extensible mechanism for modulating the structural and functional properties of proteins [1,2,3]. Among the families of enzymes that catalyze this important covalent modification event, the most prolific by far are the so-called “typical” eukaryotic protein kinases (ePKs) [4]. Typical ePKs are found in all eukaryotes and most bacterial organisms [5,6,7,8]. In *Homo sapiens*, typical ePKs account for >90% of the approximately five hundred members of the human “kinome” [9].

What factors underly the prolific nature of the typical ePK paradigm? When and for what purposes did the regulatory potential inherent in this protein kinase family first become manifest? Although the first ePKs emerged prior to the divergence of the *Archaea* and *Eucarya*, these ancestral ePKs most closely resembled the so-called “atypical” ePKs, such as the p53-related protein kinase and the RIO (right open reading frame) protein kinases [5,10], which comprise only a minor proportion of the human kinome [9]. Since the first available archaeal genome sequences encoded only atypical ePKs, it was initially believed that the typical ePKs emerged in the *Eucarya*, from which they subsequently spread by horizontal gene transfer to the *Bacteria* [5,7]. It was thus of considerable interest that as genome sequences continued to be accumulated, that open reading frames (ORF) encoding deduced typical ePKs were encountered in members of the *Archaea*, specifically within the creanarchaeal branch of this domain.

Intriguingly, the catalytic domains of the putative archaeal members of the typical ePK family bear a discernable resemblance to the set of typical ePKs responsible for phosphorylating eukaryotic translational initiation factor 2α (eIF2α). In the *Eucarya*, the eIF2α kinases phosphorylate and inactivate eIF2α in response to indicators of stress such as amino acid limitation, viral infection, unfolded proteins, *etc.* [11,12,13]. Herein we describe the basic properties of a typical ePK from the *Archaea*, SsoPK4 from the extreme hyperthermophile *Sulfolobus solfataricus,* including the effects of 3',5'-cAMP and oxidized Coenzyme A, an indicator of oxidative stress, upon its catalytic activity.

## 2. Experimental Section

### 2.1. Materials

Purchased materials included chelating Sepharose fast-flow from Amersham Biosciences (Piscataway, NJ, USA); sequencing grade trypsin from Promega (Madison, WI, USA); EDTA-free protease inhibitor cocktail from Boehringer-Mannheim (Indianapolis, IN, USA); *Pfu* Turbo DNA Polymerase, BL21-CodonPlus (DE3)-RIL cells, and a Quik-Change II site-directed mutagenesis kit from Stratagene (LaJolla, CA, USA); QIAquick PCR purification and QIAprep spin miniprep kits from Qiagen (Valencia, CA, USA); expression vector pET-29b from Novagen (San Diego, CA, USA); genomic DNA from *Sulfolobus solfataricus* P2 from the American Type Culture Collection (Manassas, VA, USA); OMIX C18 pipette tips from Varian Inc. (Palo Alto, CA, USA); Vivapure C18 microspin columns from VivaScience (Hanover, Germany); and histone type II-AS from calf thymus and oxidized Coenzyme A from Sigma-Aldrich (St. Louis, MO, USA). *Escherichia coli* TOP10 cells and all oligonucleotides used in this study (Table 1) were from Invitrogen (Carlsbad, CA, USA). All radiochemicals were purchased from Perkin Elmer Life Sciences (Waltham, MA, USA). Restriction enzymes were from New England Biolabs (Beverly, MA, USA). All other reagents were from Sigma-Aldrich (St. Louis, MO, USA) or Fisher Scientific (Pittsburgh, PA, USA).

**Table 1:** Oligonucleotides used for this study.

Name	Sequence (5ʹ to 3ʹ)	Purpose
**SsoPK4/*sso3182***		
rSsoPK4 forward	AGTAATACCATG**GC**GATTAATCTGTTACAATAC	NcoI at M1
rSsoPK4 reverse	CAGTAT**GTCG**ACTCTAAAGTAGAAAAACTCCTTTAT	SalI
rSsoPK4(198–635)	TTACTAA**C**C**A**T**GG**TTCCGAGCAAAGGAATACCTAG	NcoI at L197M
rSsoPK4(284–635)	CACCAG**CC**ATG**G**TTCCGAGCAAAGGAATACCTAG	NcoI at M284
rSsoPK4(320–635)	ATTTTAG**CC**ATG**GC**GAACTGGGATCCTAAAGTATGGGTAGG	*Nco*I at M320
rSsoPK4seq.fwd.	CCGAGCAAAGGAATACCTAG	sequencing
rSsoPK4 seq. rev.	ACATCGTTTATAGATCCACC	sequencing
D476A forward	GAGGGATATGTTCACTGTG**C**TGTAAAACCTCAAAATG	D476A
D476A reverse	CATTTTGAGGTTTTACA**G**CACAGTGAACATATCCCTC	D476A
K363A forward	GGGAATTTTTATGCTCTC**GC**GATACCGTTAATAAATTAC	K363A
K363A reverse	GTAATTTATTAACGGTATC**GC**GAGAGCATAAAAATTCCC	K363A
T606A forward	GAAAATACGTGGATAAAAAT**G**CTTATCTCTTCATATCAAAAATGG	T606A
T606A reverse	CCATTTTTGATATGAAGAGATAAG**C**ATTTTTATCCACGTATTTTC	T606A
S611A forward	GGATAAAAATACTTATCTCTTCATA**G**CAAAAATGGTAGATCCGG	S611A
S611A reverse	CCGGATCTACCATTTTTG**C**TATGAAGAGATAAGTATTTTTATCC	S611A
T606D forward	GAAAATACGTGGATAAAAAT**GA**TTATCTCTTCATATCAAAAATGG	T606D
T606D reverse	CCATTTTTGATATGAAGAGATAA**TC**ATTTTTTATCCACGTATTTTC	T606D
S611D forward	GGATAAAAATACTTATCTCTTCATA**GAC**AAAATGGTAGATCCGG	S611D
S611D reverse	CCGGATCTACCATTTT**GT**CTATGAAGAGATAAGTATTTTTATCC	S611D
KKA forward	GCGTTATTATCGAGCAGAAACATAGAGCTATTAGAATTAGC	K295N/K296I
KKA reverse	GCTAATTCTAATAGCTCTATGTTTCTGCTCGATAATAACGC	K295N/K296I
KKB forward	CTATTAGAATTAGCATGTATAAACGGGTATAAGAAAGCTTG	K304I/K305N
KKB reverse	CAAGCTTTCTTATACCCGTTTATACATGCTAATTCTAATAG	K304I/K305N
KKC forward	CATGTAAAAAGGGGTATAACATAGCTTGTGAGCAGACTAAAC	K308N/K309I
KKC reverse	GTTTAGTCTGCTCACAAGCTATGTTATACCCCTTTTTACATG	K308N/K309I
*S*-tagT2A forward	GCCCAGATCTGGGTGCGCTGGTGCCAGCGG	T2A in S-tag
*S*-tagT2A reverse	CCGCGTGGCACCAGCGCACCAGATCTGGGC	T2A in S-tag
**aIF2α/*sso1050***		
aIF2α forward	GGGTTACCATG**G**TTTACAGTAGAAGCAAACTACCCTCAG	*Nco*I at M1
aIF2α reverse	CCTCATTTTCC**GTCGAC**TTTCTTAACCACACTTATATCTACG	*Sal*I
S262A forward	GAAGAAAACGTAGATATA**GCT**GTGGTTAAGAAAGTCGACAAG	S262A
S262A reverse	CTTGTCGACTTTCTTAACCACAGCTATATCTACGTTTTCTTC	S262A
S47A&S48A forward	GCCTTGGAGTGAAGTAA**C**TA**C**CAAATGGGTTAAGAATATAAGGG	S47A&S48A
S47A&S48A reverse	CCCTTATATTCTTAACCCATTTG**G**TA**G**TTACTTCACTCCAAGGC	S47A&S48A
S47T&S48T forward	GCCTTGGAGTGAAGTAAcTAcCAAATGGGTTAAGAATATAAGGG	S47T&S48T
S47T&S48T	CCCTTATATTCTTAACCCATTTGGTAGTTACTTCACTCCAAGGC	S47T&S48T

### 2.2. Standard Procedures

Protein concentrations were determined by the method of Bradford [14] using premixed reagent and a standardized solution of bovine serum albumin, both from Pierce Biotechnology, Inc. (Rockford, IL, USA). Sodium dodecyl sulfate (SDS) -polyacrylamide gel electrophoresis (PAGE) was performed as described by Laemmli [15]. All gels were stained with Coomassie Blue as described by Fairbanks *et al.* [16]. DNA sequencing was performed by the core facility of the Virginia Bioinformatics Institute.

### 2.3. Cloning and Mutagenesis

Cloning, expression and purification of recombinant proteins was performed as described previously [17]. Open reading frame (ORF) *sso3182* was amplified by PCR using 550 ng of *S. solfataricus* P2 genomic DNA as template, primers rSsoPK4 forward and rSsoPK4 reverse (Table 1), 10 pmol each, and 2.5 units of *Pfu* Turbo DNA polymerase following the manufacturer’s recommendations with the exception that the reaction was supplemented with 2.5 mM MgCl_2_. ORF *sso1050* was amplified using the identical procedure, with the exception that the primers used were aIF2α forward and aIF2α reverse (Table 1). The resulting PCR products were purified using a QIAquick PCR purification kit. Purified PCR products were cloned into either the *Nco*I/*Sal*I or *Bam*HI/*Sal*I sites (as indicated in Table 1) of the expression vector pET-29b and the resulting plasmids used to transform competent *E. coli* TOP10 cells. Several kanamycin-resistant colonies were selected and used to inoculate 3 mL portions of LB medium containing 100 µg/mL kanamycin. The cultures were incubated overnight, the cells harvested by centrifugation, and the plasmids isolated using a QIAprep spin miniprep kit according to the manufacturer’s protocols. DNA sequencing was performed to verify the presence of inserts and the fidelity of PCR amplification.

PCR products encoding N-terminally truncated versions of SsoPK4 were generated using the above procedure, with the exception that the appropriate “forward” primer was substituted for rSsoPK4 forward (Table 1). Site-directed mutagenesis was performed using mutagenic primers listed in Table 1 and a Quik-Change II site-directed mutagenesis kit according to the manufacturer’s protocol, with the exception that PCR reactions were supplemented with 2.5 mM MgCl_2_. In order to eliminate the possibility of adventitious phosphorylation of the S-tag domain introduced by the vector, the codon for the Thr residue within it was altered to that for Ala using the primers S-tagT2A forward and S-tagT2A reverse. For assessment of trans-autophosphorylation, constructs of rSsoPK4(284–635) lacking the *S*-tag were constructed by excising the insert from the original construct by incubation with *Nde*I and *Nco*I, filling in the resulting overhangs, and performing a blunt end ligation into the vector.

DNA encoding aIF2α (47T/48T/262A) was produced from aIF2α (s262A) using primers S47T&S48T forward and S47T&S48T reverse.

### 2.4. Expression and Purification of Recombinant Proteins

*E. coli* BL21-CodonPlus (DE3)-RIL cells were transformed with ~50 ng of the appropriate plasmid (see above) and cultured overnight at 37 °C, with shaking, in 5 mL of LB medium supplemented with 100 µg/mL kanamycin and 34 µg/mL chloramphenicol. The 5 mL culture was then used to inoculate 250 mL of LB medium supplemented with 100 µg/mL kanamycin, 34 µg/mL chloramphenicol, and 4 mM l-arginine. After incubating for 2 h at 37 °C with shaking, IPTG was added to a final concentration of 0.8 mM and the culture was incubated under the identical conditions for an additional 4 h. The cells were then harvested by centrifugation and stored at −80 °C until use.

Cell pellets were thawed on ice, then resuspended, by stirring, in 5 mL of 50 mM MOPS, pH 7.0, containing 150 mM NaCl, 20 mM imidazole, 250 µg/mL lysozyme, and EDTA-free protease inhibitor cocktail. Next, the cell suspensions were sonicated on ice 5 times, for periods of 30 s each, using a Branson Sonic Power Co. (Plainview, NY, USA) Model W185 Sonifier Cell Disruptor set on full power. The suspension was cooled on ice for one minute between each period of sonic disruption. Following sonic disruption, cell debris and any unbroken cells were removed from the crude lysate by centrifugation at 4000× *g* for 30 min at 4 °C. The supernatant liquid was incubated at 65 °C for 20 min, then placed on ice and allowed to cool. Precipitated proteins were removed by centrifugation at 4000× *g* for 20 min at 4 °C and the supernatant liquid saved.

The supernatant liquid was applied to a 1 mL column of chelating Sepharose fast-flow that had been charged with NiCl_2_ according to the manufacturer’s protocols and equilibrated in 50 mM MOPS, pH 7.0, containing 150 mM NaCl, and 20 mM imidazole. Following the application of the protein sample, the column was extensively washed with 25 mL of 50 mM MOPS, pH 7.0, containing 150 mM NaCl, and 20 mM imidazole. Adherent proteins were eluted by applying 5 mL of 50 mM MOPS, pH 7.0, containing 150 mM NaCl, and 500 mM imidazole. The resulting eluent was then brought to 95% saturation by the slow addition of 3.25 g ammonium sulfate with constant stirring. The solution was incubated on ice for 20 min. The mixture was then centrifuged at 10,000× *g* for 20 min at 4 °C and the supernatant liquid discarded. The protein pellet was dissolved in 0.5 to 1 mL of 50 mM MOPS, pH 7.0, containing 15% (v/v) glycerol and the solution divided into aliquots and stored at –80 °C until use. A 250 mL culture typically yielded 1 to 5 mg of recombinant protein.

### 2.5. Assay of Protein Kinase Activity

Protein kinase activity was routinely assayed by the filter paper method of Corbin and Reimann [18] as modified by Lower and Kennelly [19]. The final volume of the assays was typically 50 μL. The quantity of rSsoPK4 assayed ranged from 0.1 to 5.0 µg. Both MgCl_2_ and MnCl_2_ were included at a final concentration of 5 mM each. The final concentration of [γ-^32^P]ATP was 100 μM. The specific activity of the [γ-^32^P]ATP generally ranged from 2 to 5 × 10^15^ cpm/mol. Phosphoacceptor proteins were generally present at a concentration of 0.5–1.0 µg/µL. The assay mixture was typically incubated for a period of 30 min to one hour at 65 °C in 50 mM MOPS pH 7.0. On occasion, the proteins within the assay mixture were resolved by SDS-PAGE in order to identify the specific polypeptides that were phosphorylated. Radiolabeled species were visualized using an Instant Imager from Packard (Downers Grove, IL, USA).

### 2.6. Sucrose Density Gradient Ultracentrifugation

The apparent molecular weight of rSsoPK4 was determined by sucrose density gradient ultracentrifugation via an adaptation of the method of Baxter-Gabbard [20] using gradients formed from eleven mL of 50 mM MOPS, pH 7.0, containing an average of 10% (w/v) sucrose. rSsoPK4(284–635), 90 µg in a volume of 200 µL, was layered on top of the gradient and then centrifuged for 48 h at 109,000× *g* at a temperature of 4 °C. Tubes containing the protein standards cytochrome c, ovalbumin, and bovine serum albumin—1 mg each, were run in parallel. Following centrifugation, the contents of each tube were collected as a series of fractions and protein concentrations determined by measuring OD_280_ for the standards or the Bradford assay for rSsoPK4.

### 2.7. Phosphoamino Acid Analysis

SsoPK4: Following incubation with rSsoPK4(284–635) and [γ-^32^P]ATP, the (auto)phosphorylation reaction was terminated by adding 12 µL of 4X Laemmli sample buffer and heating at 100 °C for 5 min. Proteins were then separated by SDS-PAGE and sections containing individual phosphoproteins excised and phosphoamino acid content analyzed following the method of Kamps and Sefton [21].

aIF2α: aIF2α, aIF2α (S262A), or aIF2α (S47T/S48T/S262A), 8.4 µg, were incubated for 45 min, at 65 °C in 36 µL of 50 mM MOPS, pH 7.0, containing 0.6 µg of rSsoPK4(284–635), 5 mM MgCl_2_, 5 mM MnCl_2_, and 30 µM [γ-^32^P]ATP. The specific radioactivity of the [γ-^32^P]ATP was ~3 × 10^16^ cpm/mol. At the end of the incubation period, 12 µL of 4X Laemmli sample buffer was added and the samples processed as described for SsoPK4.

### 2.8. Generation of Tryptic Peptides for MS Analysis

For ^32^P-labeled phosphoproteins from SDS-PAGE, trypsin digestion was conducted in gel as described previously [17] with the exception that the reduction and alkylation steps were omitted and the recovery of tryptic peptides was improved by extraction using 2 volumes of 0.5% (v/v) TFA followed by agitation in a sonicating water bath for 15 min. The supernatant liquid was collected by centrifugation and added to the original digest solution. The extraction process was repeated using two volumes of 0.5% (v/v) TFA containing 20% (v/v) acetonitrile, then 0.5% (v/v) TFA containing 40% (v/v) acetonitrile and finally with 0.5% (v/v) TFA containing 60% (v/v) acetonitrile and extracts pooled.

For rSsoPK4(284–635) and aIF2α, the proteins were (auto)phosphorylated as described in Section 2.7, above, with the exception that the final volume was increased to 200 µL and radiolabel was omitted. Reaction was terminated by the addition of four volumes of ice-cold methanol. The mixture was cooled at −80 °C for two hours and precipitated protein then collected by centrifugation at 12,000× *g* for 3 min. The resultant pellet was washed twice with ice-cold methanol, air dried, then resuspended in freshly prepared 100 mM ammonium bicarbonate and digested. Sequencing-grade trypsin was added and proteolytic cleavage conducted as previously described [17].

The tryptic digests were then applied to an aluminum hydroxide column following the metal oxide affinity chromatography to enrich for phosphorylated peptides as described in Section 2.8 of Wolschin *et al.* [22]. TFA, 5% (v/v) was added to the predicted phosphopeptide fraction to a final concentration of 0.5% (v/v) and the pH adjusted to below 3.0 using 98% (v/v) formic acid. The acidified mixture was applied to a set of VivaPure C18 microspin columns and processed as described by the manufacturer with the exception that the columns were subject to 3 to 5 final washes with 5% (v/v) formic acid containing 2% (v/v) methanol to remove TFA. Adherent peptides were then eluted from the column with 5% (v/v) formic acid in 95% (v/v) methanol. Dilute samples were air dried to near dryness, then resuspended in approximately 5 µL of 5% (v/v) formic acid in 50% (v/v) methanol using a sonicating water bath for 15 min.

### 2.9. Clean-Up of Tryptic Peptides Using C18 Material

Salt removal from tryptic peptides utilized either OMIX C18 tips from Varian Inc. (Palo Alto, CA, USA) for those isolated from polyacrylamide gel slices or VivaPure C18 microspin columns from VivaScience (Hanover, Germany) for those isolated using metal oxide affinity chromatography. Following incubation in a 65 °C heat block for approximately 30 min to remove the acetonitrile, samples were adjusted to 0.5% TFA using 5% TFA. Where required, the pH was adjusted to below 3 using 98% formic acid.

Both the tips and the microspin columns were used as described by their respective manufacturers except for the following modifications to remove the TFA prior to mass spectrometric analysis. Following the aqueous washes containing TFA, the tip or column was washed 3 to 5 times using 5% formic acid in 2% methanol. Peptides were recovered by elution with 5% formic acid in 95% methanol. Dilute samples were air dried to near dryness followed by resuspension of the sample in approximately 5 microliters of 5% (v/v) formic acid in 50% (v/v) methanol and sonication in a sonicating water bath for 15 min.

### 2.10. Analysis of Tryptic Peptides by Mass Spectrometry

Mass spectrometric analysis was conducted utilizing a Finnigan TSQ Quantum Ultra AM mass spectrometer (Thermo Electron Corp., West Palm Beach, FL, USA) equipped with a nano-electrospray source from Proxeon (Odense, Denmark). A few microliters of sample mixture were loaded into a metal-coated borosilicate emitter tip (Proxeon, Odense, Denmark) that was then inserted into the source head. The sample was then delivered to the mass spectrometer by applying sufficient pressure via a glass syringe connected to the source head to see liquid at the tip of the emitter. Set-up and use of the nanospray source and mass spectrometer were as recommended by their respective manufacturers. Spray voltage was typically 650 to 750 V and the capillary temperature was 200 °C. Data were acquired using the Quantum Tune Master in positive ion mode in the profile state. Typically, MS data were collected for five minutes while scanning the first quadrapole with a 0.7 peak width from 450 to 1500 *m/z* using a 1.45 s scan time.

The QualBrowser application of the Xcalibur software was used to average the data. From this averaged MS data, random multiply-charged peaks, typically the most intense, were chosen for MS/MS analysis. MS/MS data were collected for 1–2 min using a 0.7 peak width on both quadrapoles 1 and 3 using a 1.45 s scan time and a range from 50 to 1500 *m/z*. Quadrapole 2 CID gas was set at 1.5 mTorr and collision energy was adjusted manually for optimum fragmentation—typically from 25 to 45 eV. Again the QualBrowser application of Xcalibur was used to average the data and generate a peak list. The peak list was edited using Microsoft Excel to remove noise (retaining only the 300–500 most intense peaks and removing the parent ion when necessary) and Microsoft Notepad and/or Microsoft Wordpad to add the required header information for subsequent searching using Mascot and merging MS/MS data from the same sample together into one text file.

The resulting peak lists were analyzed using Matrix Science’s (Boston, MA, USA) Mascot MS/MS ions search. Searches were conducted using the default instrument setting and parameters corresponding to trypsin digestion with up to two possible missed cleavages, an average peptide mass tolerance of ±2 Da, and a fragment ion mass tolerance of ±0.8 Da. The variable modification parameters selected included phosphorylation of threonine and serine residues and oxidation of methionine. The probability based MOWSE score for individual ions is −10 × Log(P), where P is the probability that the observed match is a random event. Protein scores are derived from ions scores as a non-probabilistic basis for ranking protein hits [23]. All search results were verified manually.

For identification of the third site of autophosphorylation in rSsoPK4(284–635), 5% (v/v) was added directly to the tryptic digest to a final concentration of 0.5% (v/v) and the pH was adjusted to below 3.0 using 98% (v/v) formic acid. The acidified mixture was loaded into the autosampler of a Tempo NanoMDLC (Applied Biosystems, Foster City, CA, USA). Portions, 2 µL, of the acidified digest were loaded onto a 300 µm × 5 mm (5 micron particles with 100 angstrom pores) C18 PepMap 100 precolumn (Dionex, Sunnyvale, CA) at flow rate of 20 µL/min and the column washed for 10 min with a 95:5 mixture of solvent A, which consisted of 2% (v/v) HPLC-grade acetonitrile and 0.1% (v/v) formic acid in HPLC grade water and solvent B, which consists of 98% (v/v) HPLC-grade acetonitrile containing 0.1% (v/v) formic acid. After the precolumn was washed, it was placed in series with a 75 µm × 50 mm (5 micron particles with 300 angstrom pores) ProteoPep 2 column (New Objective Inc., Woburn, MA, USA) and the columns developed at a flow rate of 0.25 µL/min as follows: 95:5 solvent A: solvent B for 10 min; a linear increase to 80:20 solvent A: solvent B over five minutes; a linear increase to 65:35 solvent A: solvent B over a period of 15 min; a linear increase to 5:95 solvent A: solvent B over 5 min; then 95% (v/v) solvent B for two minutes before returning to the initial conditions of 5:95 solvent A: solvent B. Portions of the eluent were introduced into 4000 Q-Trap Mass Spectrometer via a MicroIonSpray II nanospray source (both Applied Biosystems, Foster City, CA, USA). The mass spectrometer program consisted of a repeating cycle starting with an enhanced MS (EMS) scan in positive mode from 400 to 1500 utilizing a 5 ms trap fill time and a scan speed of 4000 amu/s. This scan was followed by an enhanced resolution (ER) scan of the three most intense ions with an intensity above 300,000 cps to better estimate the *m/z* value of the ion and to help determine the charge state of the ion from its isotopic distribution. The ER scan used a dynamic fill time and a scan speed of 250 amu/s. Ions determined by the ER scan to possess a charge state of +2, +3 or +4 (or an indeterminate charge state) were then subject to an enhanced product ion (EPI) scan. EPI scans utilized a dynamic fill time, a scan speed of 4000 amu/s and a rolling collision energy calculated based upon the *m/z* and charge state of the ion. The mass spectrometry software, Analyst 1.4.2 (AB SCIEX, Framingham, MA, USA.), was used to generate text files containing the peak lists corresponding to the EPI scans performed by the mass spectrometer. The resulting peak lists were analyzed using Matrix Science’s (Boston, MA, USA) Mascot MS/MS ions search essentially as described above.

## 3. Results and Discussion

### 3.1. The Genome of Sulfolobus Solfataricus Encodes Three Deduced Typical ePKs

The hyperthermophilic archaeon *S. solfataricus* contains more than five hundred proteins that are phosphorylated on serine, threonine, and/or tyrosine residues [24]. Its genome [25] encodes several potential protein kinases, three of which conform to the typical ePK paradigm: ORFs *sso2291*, *sso3182*, and *sso3207* [26]. In general, “Itypical” ePKs can be distinguished from their “atypical” counterparts by (a) the presence of a lysine or arginine in subdomain VIb, indicative of specificity for serine/threonine or tyrosine residues, respectively; and (b) well-defined examples of the canonical ePK subdomains VIII, IX, X, and XI in the C-terminal protein substrate binding domain [8,10].

**Figure 1:**
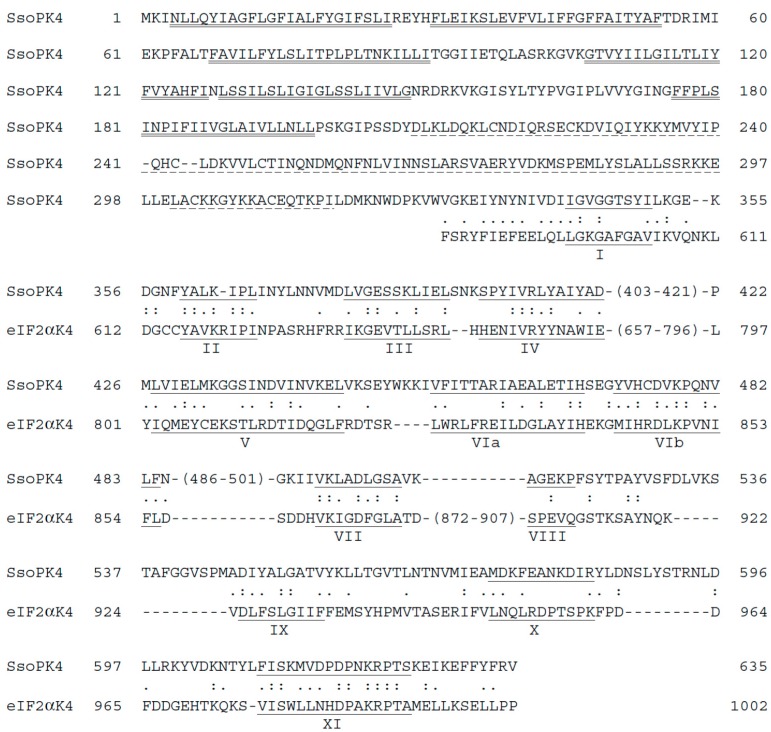
The predicted protein product of ORF *sso3182,* SsoPK4, possesses the sequence features characteristic of a ‘typical’ ePK. Shown is the predicted amino acid sequence of SsoPK4. The predicted transmembrane segments in the *N*-terminal domain are indicated by a double underline. The predicted α-helical repeat domain is indicated by a dashed underline. The sequence of the catalytic domain of murine eIF2α kinase 4, eIF2α K4 [28], is shown below that of the predicted C-terminal catalytic domain of SsoPK4. The subdomains, I–XI, characteristic of the typical ePK family are underlined and identified by an underlying roman numeral. Amino acid identities are indicated by colons (:), while similar amino acids are indicated by periods (.).

Intriguingly, the deduced catalytic domains of *sso2291, sso3182*, and *sso3207* do more than simply conform to the minimal characteristics of the typical ePK paradigm. All three exhibit substantial, ~40%, similarity over the predicted catalytic domain to a specific subfamily of typical ePKs in the *Eucarya*—those responsible for the phosphorylation and inactivation of eukaryotic translation initiation factor2α (eIF2α) (Figure 1). These similarities include the presence of an insertion between subdomains IV and V, a feature unique to the eucaryal eIF2α protein kinases [27].

**Figure 2:**
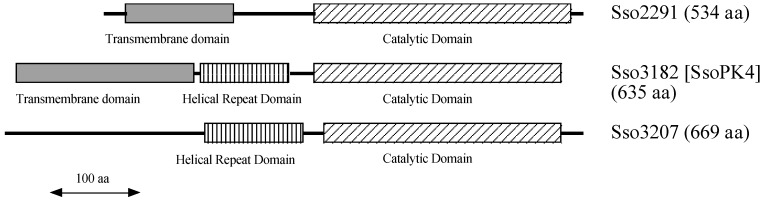
Domain organization of the deduced protein products of ORFs *sso2291*, *sso3182*, and *sso3207* from the genome of *S. solfataricus* P2 [26]. The predicted catalytic domains are indicated by diagonally hatched boxes, predicted transmembrane domains by shaded boxes, and predicted central helical repeat domains by vertically hatched boxes.

Eucaryal organisms possess from one to four distinct eIF2α protein kinases that can be differentiated from one another by the presence of a characteristic regulatory domain appended to the common catalytic core [11,12,13]. These regulatory domains render the catalytic activity of each eIF2 α kinase responsive to distinct, generally stress-related, signals. All three potential archaeal initiation factor 2α (aIF2α) kinases from *S. solfataricus* contain auxiliary domains as well (Figure 2). The N-terminal portion of the predicted protein product of ORF *sso2291*, for example, is dominated by a deduced transmembrane domain. By contrast, the deduced protein product of ORF *sso3207* contains a central, ~120 residue, predicted helical repeat domain [29] as well as an N-terminal region lacking in recognizable structural motifs. The predicted protein product of ORF *sso3182*, on the other hand, possesses a deduced N-terminal transmembrane domain as well as a central helical repeat domain.

### 3.2. The Protein Product of ORF sso3182 Exhibits Protein-Serine/Threonine Kinase Activity

ORF *sso3182* was cloned by PCR and inserted into an expression vector that encodes an N-terminal S-tag and a thirty-three residue C-terminal fusion domain that includes a polyhistidine motif. When the recombinant protein product, rSsoPK4 (*S. solfataricus* protein kinase 4, recombinant form), was expressed in *E. coli* and isolated by metal affinity chromatography, the resulting preparations contained large quantities of proteolytic breakdown products, even when large quantities of protease inhibitors were included in the lysis and purification buffers. Electrophoretically homogeneous preparations were eventually obtained by engineering and expressing a truncated version of the protein, consisting of residues 284–635, that encompassed the catalytic and central helical repeat domains. Purified rSsoPK4(284–635) sedimented as a monomer during sucrose density gradient ultracentrifugation.

**Figure 3:**
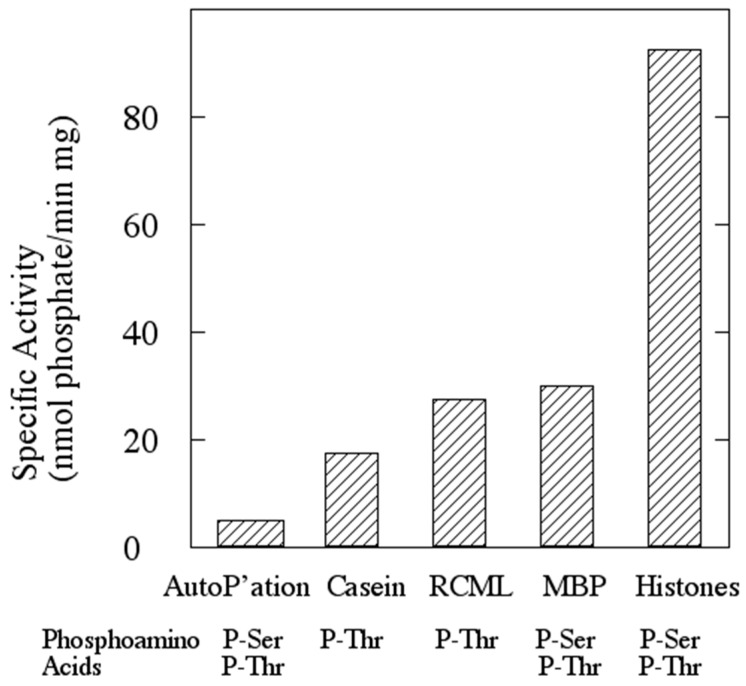
rSsoPK4 phosphorylates exogenous proteins *in vitro.* Full-length rSsoPK4 was assayed for protein kinase activity toward the indicated proteins as described under Experiment Section. Shown is the rate of incorporation of [^32^P]phosphate into TCA-precipitable proteins. Below the graph, the phosphoamino acid(s) detected from an acid hydrosylate of the protein are given. RCML, reduced, carboxamidomethylated and maleylated lysozyme; MBP, myelin basic protein.

When incubated with [γ-^32^P]ATP and exogenous proteins such as mixed histones, myelin basic protein (MBP), casein, or reduced carboxyamidomethylated and maleylated lysozyme in the presence of a metal ion cofactor; rSsoPK4 catalyzed the transfer of anywhere from 0.2 to 0.9 moles of [^32^P]phosphate per mole of protein to each (Figure 3). Mutagenic alteration of either of two predicted essential residues, Lys_363_ in subdomain II or Asp_476_ in subdomain VIb [30], to alanine each resulted in an inactive protein product. Mutagenic alteration of a non-conserved residue within subdomain IIII, Lys_382_, to alanine yielded an active protein kinase. This behavior confirmed that rSsoPK4 was the source of the protein kinase activity detected in our assays and buttressed the annotation of SsoPK4 as a typical ePK.

**Figure 4:**
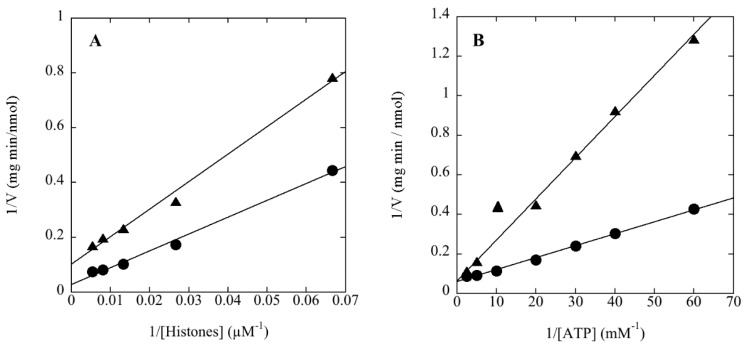
Phosphorylation of mixed histones by rSsoPK4 follows simple Michaelis-Menten kinetics. rSsoPK4(284–635), 1.8 µg, was assayed for protein kinase activity toward mixed histones as described in Experiment Section. Panel **A** shows a Lineweaver-Burk Plot in which the concentration of [γ-^32^P]ATP was fixed at 100 µM while the concentration of mixed histones was varied as indicated. Panel **B** shows a Lineweaver-Burk Plot in which the concentration of mixed histones was fixed at 70 µM, while the concentration of [γ-^32^P]ATP was varied as indicated. On both plots, the circles and triangles indicate the results obtained in the absence or presence, respectively, of 55 µM 3ʹ,5ʹ-cAMP.

Phosphoamino acid analysis of revealed that rSsoPK4(284–635) phosphorylated itself as well as the aforementioned exogenous substrate proteins exclusively on threonine or threonine and serine residues (Figure 3), behavior consistent with the presence of a lysine in subdomain VIb [31]. Catalytic activity was greatest at pH 6.8. Mn^2+^ was by far the most effective divalent metal ion cofactor, a preference that mirrors the behavior of several atypical ePKs, as well as the sole PPP-family protein phosphatase, from *S. solfataricus* [19,32,33]. rSsoPK4(284–635) exhibited Michaelis-Menten kinetic behavior toward mixed histones (Figure 4A) and MBP. The estimated Michaelis constants for rSsoPK4(284–635) toward mixed histones were V_max_, 16 nmol/min mg, and K_m_, 220 µM. The K_m_ for ATP was 100 µM (Figure 4B). V_max_ and K_m_ for MBP were 3.7 nmol/min mg and 145 µM (Table 2).

**Table 2:** Summary of kinetic constants determined during this study. K_m_ values are for the indicated protein substrates (The K_m_ for ATP was determined to be 100 µM using mixed histones as protein substrate).

Protein Kinase	Protein Substrate	CoAS-SCoA (1 mM)	V_max_ (nmol/min mg)	K_m_ (µM)
rSsoPK4(284–635)	Mixed histones	−	16	220
rSsoPK4(284–635)	MBP	−	3.7	145
rSsoPK4(284–635)	MBP	+	4.8	48
rSsoPK4(284–635)	aIF2α	−	4.2	48
rSsoPK4(284–635)	aIF2α	+	2.9	6
rSsoPK4(284–635) (T_592_D/T_606_D/S_611_D)	MBP	−	4.2	17
rSsoPK4(284–635) (T_592_D/T_606_D/S_611_D)	aIF2α	−	1.9	4

### 3.3. rSsoPK4(284–635) Phosphorylates aIF2α in Vitro

The core components of the translation initiation factor 2 complex are conserved across all three phylogenetic domains [34,35,36]. Since the α-subunit of this complex constitutes the physiological target of eucrayal eIF2α kinases, we cloned and expressed the protein product of ORF *sso1050,* which encodes the archaeal homologue of eIF2α, aIF2α, from *S. solfataricus.* rSsoPK4(284–635) phosphorylated recombinant aIF2α on serine and threonine *in vitro* with an estimated V_max_ of 4.2 nmol/min mg and a K_m_ of 48 µM (Table 2).

**Figure 5:**
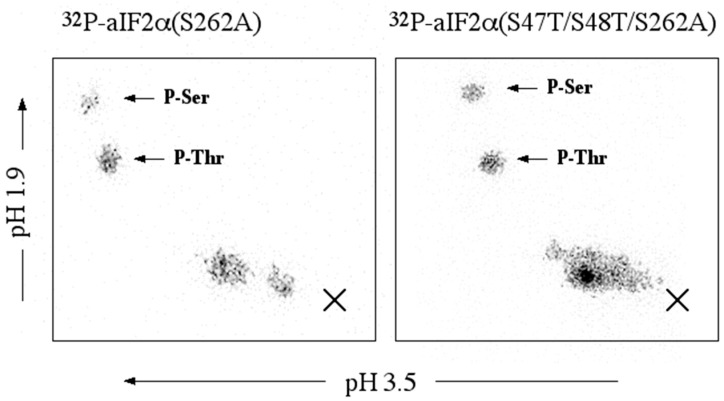
Phosphoamino acid analysis of mutagenically-altered variants of aIF2α. Variants of aIF2α in which the phosphoacceptor serine residue, Ser_262_, was replaced by alanine (aIF2α (S262A), LEFT), or in which Ser_262_ was replaced by Ala and both Ser_47_ and Ser_48_ were replaced by threonine (aIF2α (S47T/S48T/S262A), RIGHT) were incubated with rSsoPK4(284–635) in the presence of [γ-^32^P]ATP. The proteins were then resolved from one another by SDS-PAGE, the regions of the gel containing variants of aIF2α excised, and their phosphoamino acid content analyzed by 2D thin-layer electrophoresis (TLE) at pH 1.9 and pH 3.5 as described in Experiment Section. Shown are the autoradiograms of the TLE plates with identity of any comigrating phosphoamino acid standards identified using arrows and the origin marked with an X.

LC-MS-MS analysis of a tryptic digest of phosphorylated aIF2α identified two sites of phosphorylation, Thr_184_ and Ser_262_ (Table 3). However, no peptides were detected in which either Ser_47_, which corresponds to the conserved phosphorylation site on eucrayal eIF2α, Ser_51_ [37], or the immediately adjacent residue, Ser_48_, were phosphophorylated. As LC-MS-MS generally does not detect all potential peptides derived from a given protein, we turned to an alternative approach to ascertain whether aIF2α was phosphorylated at serine residues additional to Ser_262_. When aIF2α(S262A), a version of aIF2α in which Ser_262_ was substituted by a non-phosphorylatable alanine residue, was phosphorylated using rSsoPK4(284–635), phosphoamino acid analysis revealed the presence of both P-Thr and P-Ser. However, the relative level of P-Ser appeared to diminish by roughly 50% over aIF2α. To determine whether Ser_47_ or the adjacent residue, Ser_48_, could account for the P-Ser in phospho-aIF2α(S262A), site-directed mutagenesis was used to generate a new version of this protein in which Ser_47_ and the immediately adjacent residue, Ser_48_, were replaced by the alternative phosphoacceptor amino acid, threonine: aIF2α(S47T/S48T/S262A). If either Ser_47_ or Ser_48_ were the source of the remaining P-Ser, then it would be expected that replacing them with Thr would yield a protein that was phosphorylated exclusively on threonine. However, when aIF2α(S47T/S48T/S262A) and aIF2α(S262A) were phosphorylated in parallel using rSsoPK4(284–635) and [γ-^32^P]ATP, subsequent analysis of their phosphoamino acid content yielded P-Thr and P-Ser (Figure 5).

**Table 3:** Sequence analysis of phosphopeptides isolated from aIF2α following phosphorylation by rSsoPK4(284–635) *in vitro.* aIF-2α was phosphorylated by SsoPK4 *in vitro* and treated with trypsin as described in Experiment Section. Tryptic peptides were analyzed my LC-MS-MS and deduced phosphopeptides fragmented by collisionally-induced dissociation in order to determine their sequence. The fragment ions matched those predicted for a monophosphorylated tryptic peptide having the sequence MSGLIpT_184_VR (Phosphopeptide #1) and IGKEENVDIpS_262_VVK (Phosphopeptide #2). Shown are the *m/z* values of the fragment ions obtained, the calculated mass differences between them (assuming z = +1), and the amino acid residue or combination of residues possessing the corresponding mass.

Phosphopeptide (z, *m/z*)	y-ion (*m/z*)	Δm (Da)	Predicted Residue(s)
#1 ( z = +2, *m/z* = 478.7)	727.3	132.1 *	M
	640.5	86.8	S
	583.5	57.0	G
	470.3	113.2	I/L
	357.2	113.1	I/L
	274.3	82.9	2-Aminodehydrobutyrate
	n.a.	274.3	V + R
#2 ( z = +2, *m/z* = 755.3)	1114.4	298.2 *	I/L + Q/K + G
	984.5	129.9	E or M **
	855.2	129.3	E
	741.5	113.7	N
	642.5	99.0	V
	527.5	115.0	D
	414.2	113.3	I/L
	345.2	69.0	Dehydroalanine
	246.1	99.1	V
	n.a.	246.1	V + Q/K

* Determined relative to predicted mass of phosphopeptide following neutral loss of phosphate; ** Δm was midway between predicted values for E (129 Da) and M (131 Da).

### 3.4. rSsoPK4(284–635) Catalyzes Its Own Phosphorylation

Both full-length rSsoPK4 and rSsoPK4(284–635) catalyzed autophosphorylation on both serine and threonine residues. Curiously, the level of phosphate incorporation into rSsoPK4(284–635) increased many-fold when an exogenous phosphoacceptor substrate, MBP, was present in addition to [γ-^32^P]ATP. Initial LC-MS-MS analyses of a tryptic digest of rSsoPK4(284–635) identified two closely clustered autophosphorylation sites, Thr_606_ and Ser_611_ (Table 4). Later analyses revealed the presence of a third site located on either Ser_591_ or Thr_592_ (Table 4).

All three autophosphorylation sites are located in the region between subdomains X and XI of the catalytic domain, rather than in the canonical activation loop (Figure 1).

**Figure 6:**
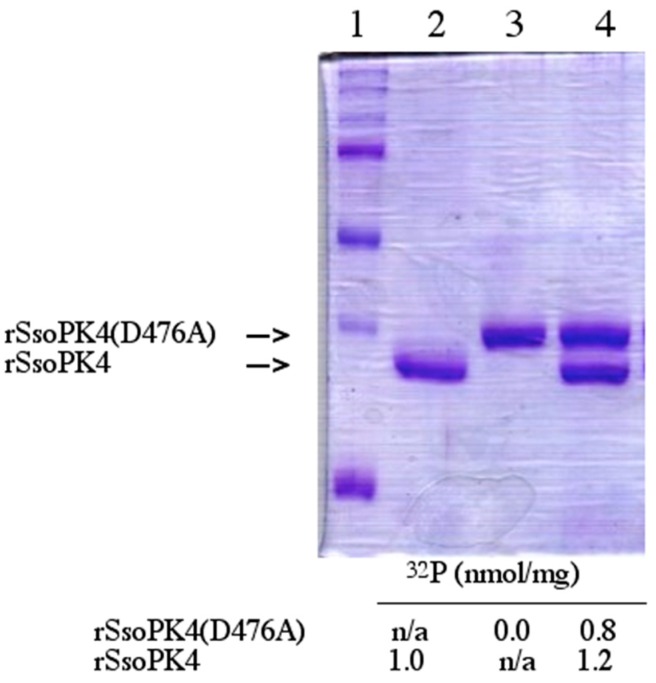
Autophosphorylation of rSsoPK4 occurs *in trans.* rSsoPK4(284–635) and a catalytically inactive form of the enzyme, rSSoPK4(D476), 4 µg each, were incubated separately or together with [γ-^32^P]ATP in a volume of 50 µl as described in Experiment Section. rSsoPK4(D476A) was expressed with an N-terminal S-Tag^TM^ (Novagen, San Diego, CA, USA) domain present to increase its molecular mass relative to rSsoPK4(284–635). Portions, 40 µL, of each reaction mixture were applied to an SDS-polyacrylamide gel. Shown below is the Coomassie stained gel. Lane 1 contains protein standard, Lane 2 contains rSsoPK4(284–635), Lane 3 contains rSsoPK4(D476A), and Lane 4 contains the mixture of rSsoPK4(284–635) and rSsoPK4(D476A). Sections of the gel containing each protein were excised and their ^32^P content analyzed by liquid scintillation counting. Shown below are the results reported in nmol of [^32^P]phosphate per mg of protein.

In order to determine whether autophosphorylation could take place *in trans,* a catalytically inactive form of the enzyme, rSsoPK4(284–635)D476A, was constructed, expressed, and purified. In this variant, the catalytically essential aspartic acid residue in subdomain VIb, Asp_476_, was substituted by alanine to form rSsoPK4(D476A). In order to insure that the inactive variant rSsoPK4(284–635)D476A could be separated from active rSsoPK4(284–635) by SDS-PAGE, a version of rSsoPK4(284–635) in which a 15-residue the S-tag domain was absent was engineered, expressed, and purified. As expected, no autophosphorylation could be detected when rSsoPK4(284–635)D476A was incubated with [γ-^32^P]ATP. However, when it was incubated with catalytically active rSsoPK4(284–635) and [γ-^32^P]ATP, radioactive phosphate was incorporated into both rSsoPK4(284–636)D476A and active rSsoPK4(284–635) itself (Figure 6). These results indicate that rSsoPK4(284–636)D476A had been trans-autophosphorylated by rSsoPK4(284–635).

**Table 4:** Sequence analysis of phosphopeptides isolated from rSsoPK4(284–635) following autophosphorylation *in vitro.* rSsoPK4(284–635) was incubated with ATP and digested with trypsin as described in Experiment Section. Tryptic peptides were analyzed my LC-MS-MS and the two deduced phosphopeptides were fragmented by collisionally-induced dissociation in order to determine their sequence. The fragment ions match those predicted for a monophosphorylated tryptic peptide having the sequence YLDNSLYS_591_TR that is phosphorylated on either Ser_591_ or Thr_592_ (Phosphopeptide 1) and a diphosphorylated peptide having the sequence YVDKpT_606_YLFIpS_611_K (Phosphopeptide 2). Shown are the *m/z* value of the fragment ions obtained, the calculated mass differences [Δm] between them (assuming z = +1), and the amino acid residue or combination of residues whose predicted mass most closely matches Δm. The abbreviation n.d. indicates the particular ion was not detected.

**Phosphopeptide (z, *m/z*)**	**b-ion (*m/z*)**	**y-ion (*m/z*)**	**y-P_i_ ion (*m/z*)**	**Δm (Da)**	**Predicted Residue(s)**
#1 (z = +2, *m/z* = 656.4)	n.d.	1148.5	1050.5	164.3	Y
	277.1	1035.3	937.4	113.2	I/L
	392.3	920.4	822.4	115.1	D
	506.2	806.4	708.4	114.0	N
	593.3	719.4	621.4	87.0	S
	706.3	606.2	508.3	113.1	I/L
	n.d.	443.1	345.3	163.1	Y
	n.d.	n.d.	n.d.	443.2	p(S + T + R)
	n.d.	n.d	n.d.	n.a.	n.a.
	n.d.	175.0	n.d.	175.0	R
**Phosphopeptide (z, *m/z*)**	**a-ion (*m/z*)**	**b-ion (*m/z*)**	**y-ion (*m/z*)**	**Δm (Da)**	**Predicted residue(s)**
#2 (z = +2, *m/z* = 826.0)	136.1		1291.7	136.1	Y
	235.1	n.d.	1192.6	99.1	V
	n.d.	378.2	1077.6	115.0	D
	n.d.	506.3	949.5	128.1	Q/K
	n.d.	620.3	835.5	114.0	N
	n.d.	n.d.	752.4	83.1	2-Amino-dehydrobutyrate
	n.d.	n.d.	589.4	163.0	Y
	n.d.	n.d.	476.3	113.1	I/L
	1098.6	1126.6	329.2	147.1	F
	1211.6	n.d.	216.1	113.1	I/L
	n.d.	n.d.	n.d.	216.1	Dehydroalanine + K/Q

### 3.5. rSsoPK4 Is Inhibited by 3ʹ,5ʹ-cAMP *in Vitro*

The central helical repeat domain of SsoPK4 bears a faint resemblance to the S-adenosyl-methionine binding motifs in other proteins. We therefore asked whether the activity of SsoPK4 might be affected by S-adenosyl-methionine or nucleotide-containing compounds. rSsoPK4(284–635) was assayed in the presence of a wide range of nucleotides, amino acids, coenzymes, *etc.* Only a handful of these compounds perturbed the activity of rSsoPK4 when present at a concentration of 100 µM, including 3ʹ-AMP, 2ʹ,3ʹ-cAMP, 3ʹ,5ʹ-cAMP, ADP, adenosine phosphosulfate, and 5ʹ-AMP (Table 5). Further investigation revealed that only three of the compounds exhibited sub-millimolar IC_50_ values under the assay conditions employed: ADP (~190 µM), adenosine phosphosulfate (~190 µM), and 3ʹ,5ʹ-cAMP (~50 µM). Analysis of the most potent inhibitor, 3ʹ,5ʹ-cAMP, revealed that it acted noncompetitively with respect to the phosphoacceptor substrate mixed histones (Figure 4A) and competitively with respect to the phosphodonor substrate ATP (Figure 4B), with a calculated K_i_ of 23 µM.

**Table 5:** Effect of nucleotides on the activity of rSsoPK4. Catalytic activity of rSsoPK4 was measured using MBP as phosphoacceptor substrate. All effectors were tested at a concentration of 100 µM. Compounds were designated as inhibitors (Y) if they were observed to stimulate or reduce, respectively, catalytic activity by greater than 20% relative to controls lacking added effectors at a concentration of 100 µM. Where a change of ±20% or less in enzyme activity was observed, the compound was designated as non-inhibitory (N). For those compounds that produced apparent inhibition during the initial screen, further analyses were conducted to estimate their IC_50_, values, which are reported in the third column. n.d. = not determined.

Nucleotide	Inhibitor	IC_50_ (µM)	Nucleotide	Inhibitor	IC_50_ (µM)
ADP	Y	190	NAD^+^	N	n.d.
5'AMP	Y	920	NADH	N	n.d.
3ʹ-AMP	Y	2100	NADP^+^	N	n.d.
5ʹ-dAMP	N	n.d.	NADPH	N	n.d.
GTP	N	n.d.	FAD^+^	N	n.d.
5ʹ-GMP	N	n.d.	Thiamine-PP_i_	N	n.d.
5ʹ-CMP	N	n.d.	CoASH	N	n.d.
5ʹ-UMP	N	n.d.	Acetyl-CoA	N	n.d.
Phosphoadenosine phosphosulfate	N	n.d.	S-Adenosyl-Methionine	N	n.d.
Adenosine phosphosulfate	Y	190	S-Adenosyl-homocysteine	N	n.d.
3ʹ,5ʹ-cAMP	Y	50	2ʹ,3ʹ-cAMP	Y	1550

### 3.6. Residues 284-319 Are Necessary for Inhibition by 3',5'-cAMP

**Figure 7:**
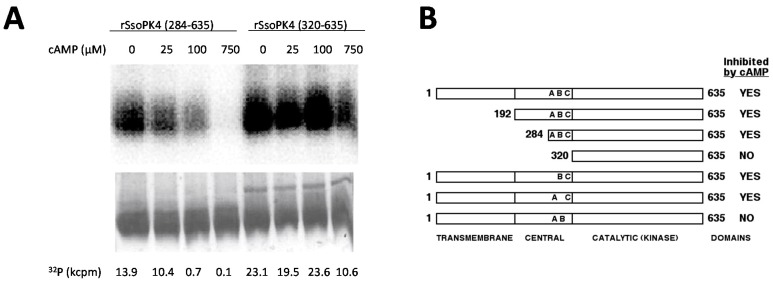
The central domain is necessary for inhibition by 3ʹ,5ʹ-cAMP. Full length, truncated, and mutagenically-altered versions of SsoPK4 were constructed, expressed, purified, and assayed for protein kinase activity using mixed histones as substrate in the presence of the indicated concentrations of 3ʹ,5ʹ-cAMP. PANEL **A** shows the electronic autoradiogram (top) of a section of a Coomassie-stained gel (bottom) on which the phosphorylated histones were isolated by SDS-PAGE following incubation with [γ-^32^P]ATP and rSsoPK4 that had been N-terminally truncated at either residue 283, rSsoPK4(284–635), or 319, rSsoPK4(320–635). The quantity of ^32^P radioactivity associated with the major Coomassie stained protein band in each lane, in cpm × 10^3^ (kcpm), is listed below. PANEL **B** shows a schematic diagram of the all the forms of SsoPK4 tested and their sensitivity to inhibition (>50% threshold) by 100 µM 3ʹ,5ʹ-cAMP. The positions of the three lysine dyads altered by site directed mutagenesis are indicated by letters: A, K_295_K_296_; B, K_304_K_305_; and C, K_308_K_309_. Omission of a letter indicates that both lysine residues had been replaced by NI (A and C) or IN (B).

We next asked whether the presence of the transmembrane and/or central helical repeat domains was required for the inhibition of rSsoPK4’s protein kinase activity by 3ʹ,5ʹ-cAMP. While deletion residues 1–319, which included both the membrane spanning domain and central helical repeat domain, had little effect upon the protein kinase activity of rSsoPK4, rSsoPK4(320-635) was rendered insensitive to 3',5'-cAMP (Figure 7). However, restoration of the C-terminal portion of the central helical repeat domain, specifically residues 284–319, proved sufficient to restore sensitivity to 3ʹ,5ʹ-cAMP.

One of the more conspicuous features of the residue 284–319 region was the presence of three lysine dyads: K_295_K_296_, K_304_K_305_ and K_308_K_309_. Given the anionic character of 3ʹ,5ʹ-cAMP, we asked whether presence of any or all of these lysine dyads was required to maintain sensitivity to 3ʹ,5ʹ-cAMP. Constructs encoding full-length rSsoPK4 were prepared in which the lysine dyads were systematically replaced by asparagine followed by isoleucine (K_295_N/K_296_I, K_308_N/K_309_I) or isoleucine followed by glutamine (K_304_I/K_305_N). The recombinantly-expressed products of all three constructs were catalytically active. While the activity of the mutagenically-altered forms in which the K_295_K_296_ or K_304_K_305_ dyads had been altered could still inhibited by the addition of 3ʹ,5ʹ-cAMP (100 µM), the activity of the K_308_N/K_309_I variant was insensitive to this cyclic nucleotide. These results are summarized in Figure 7B. Taken together with the results obtained from the various N-terminally truncated versions of the protein kinase, it appears that while 3ʹ,5ʹ-cAMP is a competitive inhibitor with respect to ATP, it acts allosterically, at a site distinct from the enzyme’s active site.

### 3.7. rSsoPK4(284–635) Is Activated by Oxidized CoA

**Figure 8:**
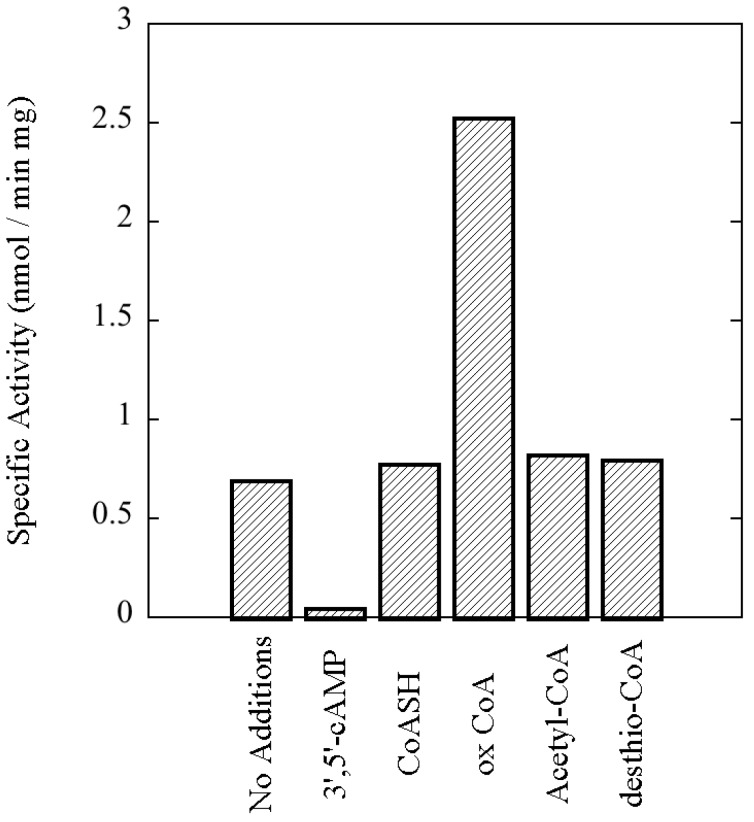
rSsoPK4(284–635) is activated by oxidized CoA. rSsoPK4(284–635), 1.5 µg, was assayed for phosphotransferase activity toward MBP, 0.6 µg/µL, in the presence of the indicated compounds, each present at a concentration of 1 mM. Shown is the specific activity of the enzyme in the presence of each of the indicated compounds. The experiment shown is representative of three analyses.

**Figure 9:**
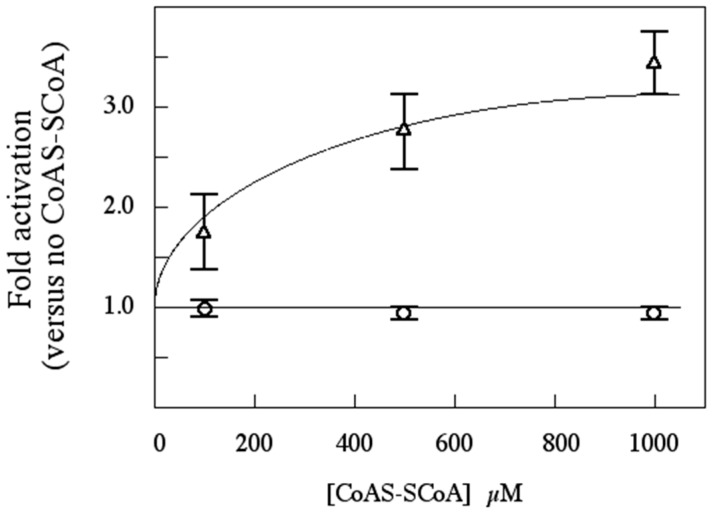
CoAS-SCoA activates rSsoPK4(284–635), but not rSsoPK4(284–635) T592D/T606D/S611D, in a concentration-dependent manner. The activity of rSsoPK4(284–635) (OPEN TRIANGLES) and rSsoPK4(284–635) T592D/T606D/S611D (OPEN CIRCLES) were assayed, in duplicate, in the presence of the indicated concentrations of CoAS-SCoA using MBP as substrate. Shown is a plot of the activity relative to that measured in the absence of CoAS-SCoA, which was normalized as 1.0, plus or minus mean average deviation.

It was noted during our screen of potential nucleotide effectors that variable results were obtained when reduced CoA was tested. While little or no effect was observed in some experiments, at other times increases in activity beyond that attributable to normal experimental variability were evident. We hypothesized that the variability might be due to the oxidation of a portion of the CoA during the course of our assays, which are conducted at a temperature, 65 °C, that approaches the hyperthermophilic conditions, 70–85 °C, of *S. solfataricus*’ natural environment. Therefore, several different CoA derivatives were tested, including desthio-CoA and oxidized CoA (CoAS-SCoA). The latter was observed to stimulate protein kinase activity toward MBP several-fold (Figure 8). Activation was concentration dependent (Figure 9). A similar pattern was observed with multiple preparations of rSsoPK4(284–635), and if either aIF2α or autophosphorylation were used to monitor protein kinase activity instead of MBP (data not shown). Oxidized glutathione had no effect on enzyme activity, suggesting that the stimulatory effect of oxidized CoA was not simply the result of a change in the redox state of some functional group on the enzyme.

**Figure 10:**
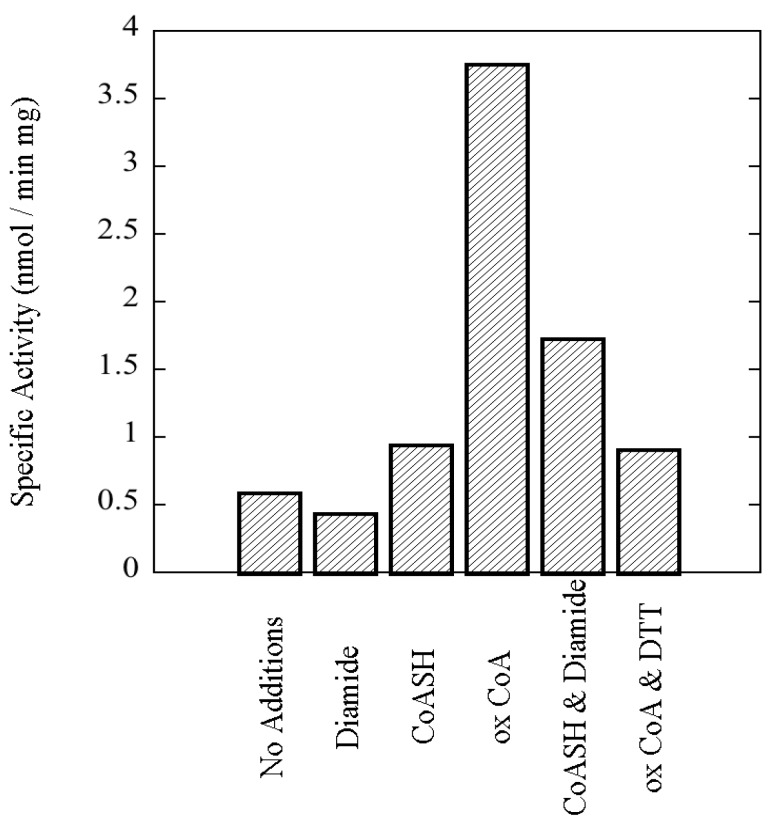
Oxidation-reduction modulates the capacity of oxidized CoA to stimulate the activity of rSsoPK4(284–635). rSsoPK4(284–635), 0.25 µg, was assayed for phosphotransferase activity toward MBP, 0.5 µg/µL, for 60 min at 65 °C in the presence of the indicated factors. The latter include diamide (Diamide), 1 mM; reduced CoA (CoASH), 1 mM; oxidized CoA (ox CoA), 1 mM; reduced CoA that had been preincubated for 30 min with an equimolar quantity of diamide (CoASH & Diamide), or 1 mM; oxidized CoA that had been preincubated for 30 min with an equimolar quantity of DTT (ox CoASH & DTT). Shown is the result of a representative experiment (*n* = 3) describing the specific activity of the enzyme as measured in the presence of the listed compounds.

To verify that the activating factor was indeed oxidized CoA, and not some contaminating species, we exploited the reversibility of disulfide bond formation. If oxidized CoA was the activator, then treating reduced CoA with diamide, a sulfhydryl-oxidizing agent [38], should transform that innocuous compound into an activator. Conversely, reduction of oxidized CoA should abrogate its inhibitory effects. As shown in Figure 10, when put to the test both predictions were fulfilled. The transformation of the bulk of the reduced CoA to oxidized CoA upon treatment with diamide, and the reduction of the bulk of the oxidized CoA by DTT, were both verified by mass spectral analysis (Data not shown). Kinetic analyses revealed that oxidized CoA increased catalytic efficiency by lowering the K_m_ for aIF2α, SsoPK4’s presumed physiologic substrate, from ~48 µM in the absence of oxidized CoA to ~6 µM in its presence, a shift of ~8-fold (Table 2). By contrast, oxidized CoA had little or no effect on V_max_, 4.2 nmol/min mg without *versus* 2.9 nmol/mg min in its presence (Table 2).

As mentioned earlier, similar results were obtained using MBP as phosphoacceptor substrate, indicating that the stimulatory effect of CoAS-SCoA is enzyme- rather than substrate-mediated. The catalytic parameters for the phosphorylation of aIF2α by activated rSsoPK4(284–635) were fairly comparable to those describing the phosphorylation of eIF2 α by homologous eucaryal eIF2α protein kinases*.* For example, when activated by dsRNA, the double-stranded-RNA-dependent eIF2α protein kinase (PKR) from phosphorylated eIF2α with a reported V_max_ of 3.3 nmol/min mg and a reported K_m_ of 0.38 µM [39]. K_m_ values reported for other eucaryal eIF2 α protein kinases toward eIF2α ranged from 0.17–2.6 µM [40,41,42]. In these latter studies, V_max_ values were either left unreported or were not be calculated due to the heterogeneity of the available enzyme preparations, which were isolated from biological sources such as reticulocytes or ascites cells.

### 3.8. Functional Impact of Autophosphorylation

Autophosphorylation is an obligate step in the activation of eucaryal eIF2α kinases by effectors such as dsRNA (PKR) or uncharged tRNAs (GCN2) [27,43,44,45,46,47,48]. We reasoned that if the activation of SsoPK4 by oxidized CoA proceeded by a mechanism involving autophosphorylation, then mutagenically-altered variants of rSsoPK4(284–635) in which one or more of the residues modified by autophosphorylation were substituted with nonphosphorylatable amino acid residues should no longer be sensitive to oxidized CoA. As anticipated, substitution of Thr_606_ and Ser_611_ or Thr_592_, Thr_606_, and Ser_611_ with aspartate residues produced forms of the enzyme, rSsoPK4(284–635)T606D/S611D and rSsoPK4(284–635) T592D/T606D/S611D, whose activity was no longer sensitive to oxidized CoA (Figure 9).

Aspartate was selected as the substitute amino acid residue in an attempt to mimic the autophosphorylated state of the SsoPK4. Examination of a pseudo-autophosphorylated form of rSsopK4, rSsoPK4(284–635)T592D/T606D/S611D, revealed that its kinetic behavior mirrored that exhibited by rSsoPK4(284–635) in the presence of CoAS-SCoA rather than in its absence. Specifically, the estimated V_max_ and K_m_ values toward aIF2α were 1.9 nmol/min mg and 4 µM, respectively, as compared to 4.2 nmol/min mg and 48 µM for the rSsoPK4(284–635) minus activator and 2.9 nmol/min mg and 6 µM in the presence of 1 mM CoAS-SCoA (Table 2). Similar results were obtained when MBP was used as phosphoacceptor substrate (Table 2).

### 3.9. Is SsoPK4 an aIF2α Protein Kinase?

A definitive answer to this question cannot be reliably extrapolated from the results of this exclusively *in vitro* study. Points in favor of an affirmative answer include the high degree of sequence similarity (~40%) shared between the catalytic domains of SsoPK4 and eIF2α protein kinases relative to the extreme phylogenetic separation of their host organisms. The common capacity to be activated by indicators of cellular stress offers a second provocative parallel between eucaryal eIF2α protein kinases [11,12,13,49,50] and rSsoPK4. It may also be noteworthy that, in the presence of activators, the archaeal enzyme phosphorylated aIF2α *in vitro* at a rate and with an apparent substrate affinity comparable to that with which eIF2α protein kinases phosphorylated eIF2α.

On the other hand, we uncovered no evidence for conservation between the sites at which rSsoPK4(284–635) phosphorylated aIF2α *in vitro* and that universally targeted by eIF2α protein kinases on eIF2α, Ser_51_ [11,12,13]. Instead of the presumed canonical residue, Ser_47_, LC-MS-MS revealed that rSsoPK4(284–635) phosphorylated aIF2α at two other sites, Ser_262_ and Thr_184_. While experiments with mutagenically-altered forms of aIF2α suggest the presence of an additional site of serine phosphorylation, it does not appear to be either Ser_47_ or its neighbor, Ser_48_.

Is it possible that phosphorylation of either Thr_184_ or Ser_262_ could alter the functional properties of aIF2α in the same way that phosphorylation at Ser_51_ does for eIF2α? Of the two, only Thr_184_ is conserved across the *Archaea* (Table 6). A growing body of evidence suggests that, even when enzyme-substrate relationships are conserved for protein kinases over long evolutionary distances, functional conservation does not necessarily extend to the sites at which downstream targets are phosphorylated [51,52,53,54,55]. These examples of phosphorylation site plasticity presumably reflect the inherent potency and versatility of the phosphoryl group as an agent for perturbing protein structure [2,3]. So *a priori*, failure to detect phosphorylation of Ser_47_ is insufficient to rule out regulation of aIF2α function by phosphorylation, or to automatically eliminate SsoPK4 as a potential physiologic aIF2α protein kinase.

**Table 6:** Alignment of Thr_184_ of aIF2α from *S. solfataricus* with other serine or threonine residues within the deduced sequences of homologous archaeal proteins. Serine and threonine residues that align with Thr_184_ from *S. solfataricus* aIF2α are highlighted using bold type.

Archaeon	Sequence	Position of Residue in Bold
*Sulfolobus solfataricus*	glitvrtnep	184
*Sulfolobus acidocaldarius*	dvislrtidp	190
*Sulfolobus tokadaii*	eivtlrssdp	190
*Pyrobaculum aerophilum*	kivsvegdgv	191
*Pyrobaculum islandicum*	kavsvegdga	191
*Aeropyrum pernix*	tlrsmagdgv	203
*Thermoproteus neutrophilus*	kavsvegdga	191
*Igniccocus hospitalis*	ilqsfapdgv	191

### 3.10. Does 3',5ʹ-cAMP Regulate SsoPK4 in Vivo?

The catalytic activity of rSsoPK4(284–636) is inhibited by, 3ʹ,5ʹ-cAMP, with an estimated K_i_ of **~**23 µM. What is the likelihood that this classic eucaryal second messenger regulates SsoPK4 *in vivo*? Genomics indicate that ORFs encoding deduced adenylyl cyclases [56] and phosphodiesterases [57] are present throughout the *Archaea*. Moreover, many of these are components of likely transmembrane receptor proteins [56]. cAMP has thus far been detected in four phylogenetically and metabolically diverse members of the *Archaea: Halobacterium salinarium* [58], *Halobacterium volcanii* [59]. *Methanobacterium thermoautotrophicum* [58], and *S. solfataricus* [59]. The basal concentration of 3ʹ,5ʹ-cAMP in *H. salinarium,* which oscillates during the cell division cycle, has been estimated to be 200 µM, while the quantity in *S. solfataricus* was reported as **~**110 pmol/mg protein. If one assumes that protein content of *S. solfataricus* is **~**200 mg/mL [60], we estimate the concentration of 3ʹ,5ʹ-cAMP to be roughly 22 µM, sufficient to significantly inhibit SsoPK4.

### 3.11. Is CoAS-SCoA a Plausible Physiological Activator for SsoPK4?

The most surprising outcome of this study was the discovery that oxidized CoA, CoAS-SCoA, activated recombinantly expressed SsoPK. Activation was consistently observed using multiple preparations of rSsoPK4, with either aIF2α or MBP as substrate, and regardless of whether CoAS-SCoA was purchased or generated by oxidizing reduced CoASH in the laboratory. At first glance, CoAS-SCoA would appear to be an unlikely candidate for a signaling intermediary. However, its dimeric symmetry renders it ideal for inducing the transautophosphorylation events that underlie activation of eucaryal eIF2α [13]. It is also consistent with CoASH’s role as the major low molecular weight thiol component of the antioxidant defense systems in archaeal (and many bacterial) organisms [61,62].

Can the concentration of CoAS-SCoA in *S. solfataricus* rise to levels sufficient to activate rSsoPK4 *in vitro*? The concentration of the total coenzyme A pool in archaeal organisms reportedly ranged, depending upon growth conditions, from 180 µM in *S. solfataricus* to 860 µM in *P. furiosus* [61]. Intriguingly, when *P. furiosus* was cultured in the presence of elemental sulfur, the size of the total CoASH pool nearly doubled from 450 to 860 µM with a concomitant increase in the proportion present as CoAS-SCoA to nearly 50% [61]. The data reported for *S. solfataricus* was for cells grown without elemental sulfur. Assuming that the estimated total CoASH pool in *S. solfataricus* could become oxidized to a similar degree to that in *P. furiosus,* the concentration of CoAS-SCoA would approach or exceed 50 µM, depending upon whether total pool size increased, a level sufficient to at least partially activate SsoPK4. While further study is needed to more clearly define the range of total and oxidized coenzyme A in *S. solfataricus,* preliminary estimates suggest that CoAS-SCoA may “moonlight” as a signaling molecule in the *Archaea.*

## 4. Conclusions

### How Did S. solfataricus and Other Archaeons Acquire Typical ePKs such as SsoPK4?

The characteristics of SsoPK4 and its archaeal homologs suggest that the ePK paradigm, specifically an early eIF2α kinase-like protein, was acquired by an ancient crenarchaeon via horizontal gene transfer [63,64] from the *Eucarya.* This model provides the simplest explanation for the limited distribution of the typical ePKs amongst the *Archaea.* Moreover the similarities, however basic, between the mechanism by which oxidized CoA activates SsoPK4 and the activation mechanisms of eucaryal eIF2α kinases, particularly the employment of the unique kinase insert domain between subdomains IV and V, is more suggestive of a common, relatively mature progenitor rather than convergence. While many questions remain to be answered, SsoPK4 provides a new, strategically-positioned vehicle for investigating the origins and evolution of the ePK paradigm as a cornerstone of biological signal transduction pathways and data integration networks.
